# Transcriptome analysis of *Asparagus officinalis* reveals genes involved in the biosynthesis of rutin and protodioscin

**DOI:** 10.1371/journal.pone.0219973

**Published:** 2019-07-22

**Authors:** Tae Gyu Yi, Young Rog Yeoung, Ik-Young Choi, Nam-Il Park

**Affiliations:** 1 Department of Plant Science, Gangneung-Wonju National University, Gangneung, Republic of Korea; 2 Department of Agriculture and Life Industry, Kangwon National University, Chuncheon, Republic of Korea; Jilin University, CHINA

## Abstract

Garden asparagus (*Asparagus officinalis* L.) is a popular vegetable cultivated worldwide. The secondary metabolites in its shoot are helpful for human health. We analyzed *A*. *officinalis* transcriptomes and identified differentially expressed genes (DEGs) involved in the biosynthesis of rutin and protodioscin, which are health-promoting functional compounds, and determined their association with stem color. We sequenced the complete mRNA transcriptome using the Illumina high-throughput sequencing platform in one white, three green, and one purple asparagus cultivars. A gene set was generated by *de novo* assembly of the transcriptome sequences and annotated using a BLASTx search. To investigate the relationship between the contents of rutin and protodioscin and their gene expression levels, rutin and protodioscin were analyzed using high-performance liquid chromatography. A secondary metabolite analysis using high-performance liquid chromatography showed that the rutin content was higher in green asparagus, while the protodioscin content was higher in white asparagus. We studied the genes associated with the biosynthesis of the rutin and protodioscin. The transcriptomes of the five cultivars generated 336 599 498 high-quality clean reads, which were assembled into 239 873 contigs with an average length of 694 bp, using the Trinity v2.4.0 program. The green and white asparagus cultivars showed 58 932 DEGs. A comparison of rutin and protodioscin biosynthesis genes revealed that 12 of the 57 genes associated with rutin and two of the 50 genes associated with protodioscin showed more than four-fold differences in expression. These DEGs might have caused a variation in the contents of these two metabolites between green and white asparagus. The present study is possibly the first to report transcriptomic gene sets in asparagus. The DEGs putatively involved in rutin and protodioscin biosynthesis might be useful for molecular engineering in asparagus.

## Introduction

*Asparagus officinalis* L. has been used as a medicinal plant and widely consumed globally for a long time, making it is an economically valuable plant [1]. Extracts of asparagus have been reported to increase insulin levels, which is helpful for improving type 2 diabetes [2] and protecting liver cells [3]. Furthermore, it exhibits anti-inflammatory, antimicrobial, and anticancer effects [4].

The major functional components of asparagus are flavonoids and steroidal saponins [5]. Flavonoids have anti-allergic, anti-inflammatory, antiviral, and antioxidant activities [6]. Rutin comprises the largest proportion of flavonoids in asparagus [7]. Rutin is a glycoside of quercetin and has been reported to have antimicrobial and anti-inflammatory [8] effects. It has also been demonstrated to have beneficial effects on diabetes [9] and arthritis [8]. Additionally, it exhibits protective effects against hepatocellular toxicity [10]. Various steroidal saponins, which impart a bitter taste, have been isolated from asparagus [11],. These compounds have been reported to affect cholesterol metabolism [12] and protect the liver [13]. They have different structures and are, therefore, useful for pharmaceutical and other commercial applications [14]. The most common steroidal saponin in asparagus is protodioscin, which affects sexual function and sex hormones [15].

Asparagus can have a variety of colors, including green, white, and purple, depending on the cultivation environment and cultivar. It contains both rutin and protodioscin, and its antioxidant ability varies with color [16]. In particular, green and white asparagus varieties are formed according to the light conditions during their cultivation. Asparagus becomes green when it receives light and white when its exposure to light is blocked. This variation is associated with differences in rutin and protodioscin contents of asparagus [16]. Green asparagus has a higher content of rutin than white asparagus, while white asparagus has a higher content of protodioscin [17]. Many studies have been conducted on the antioxidant effects and functional components of asparagus [18]. However, most molecular biology studies focused on only one of the green and white characteristics [19, 20]. Therefore, in order to compare the differences in the rutin and protodioscin content of green and white asparagus, molecular biology studies are needed to compare the differences in gene expression between the two colored varieties.

The genes involved in the secondary metabolism are more diverse than those involved in the primary metabolism. The present study aimed to analyze the whole-genome transcriptome of *A*. *officinalis* and identify differentially expressed genes (DEGs) involved in the biosynthesis of rutin and protodioscin (as functional health-promoting compounds), and their association with stem color. The identification of potential candidate genes associated with the secondary metabolic pathways based on transcriptome and DEG analyses will improve our understanding of the regulation and biosynthesis of secondary metabolites [14].

The present study aimed to investigate the genetic differences between rutin and protodioscin, among the green and white varieties of asparagus.

## Materials and methods

### Plant materials

Asparagus was cultured in a farm located in Chuncheon-si, Gangwon-do, Korea. In the present study, the commonly used cultivars ‘Atlas’ (green and white), ‘Gijnlim’ (green), ‘Pacific purple’ (purple), and ‘UC157’ (green), which were grown for 5 years, were used (Table 1). Asparagus spears (20–25 cm length) were sampled for transcriptome sequencing and metabolite content analysis. The samples were washed with distilled water, immediately frozen in liquid nitrogen, and stored at −80°C for RNA extraction and metabolite content analysis.

**Table 1:** The garden asparagus samples used for secondary metabolite and RNA sequencing analysis.

Sample name	Cultivar name	Shoot color	Cultivation type to get sunshine in asparagus
Atlas_G	Atlas	Green	Open to sunshine
Atlas_W	Atlas	White	Closed by soil to sunshine
Gijnlim	Gijnlim	Green	Open to sunshine
Pacific	Pacific	Purple	Open to sunshine
UC157	UC157	Green	Open to sunshine

### RNA extraction and Illumina sequencing

Asparagus spears of ‘Atlas,’ ‘Gijnlim,’ ‘Pacific purple,’ and ‘UC157’ were frozen in liquid nitrogen and homogenized using a mortar and pestle. Each asparagus spear was individually sampled with three tissues of each line. The extracted total mRNA was pooled from each of the three biological replicate samples. Total RNA was extracted using the Ribospin Kit (GeneAll Biotechnology Co., Seoul, Korea) according to the manufacturer’s protocol. DNA digestion was performed using DNase I (Sigma, St. Louis, MO, USA). The total RNA was quantified by measuring the absorption of light at A_260_, and the quality was checked by using a microvolume UV-vis spectrophotometer (Colibri, Titertek-Berthold, Germany). The cDNA library and massive parallel sequencing reads were generated using the Illumina mRNA Sequencing Kit and high-throughput sequencing platform according to the manufacturer’s protocol (Illumina, Inc., San Diego, CA US) by a professional sequencing provider (Macrogen Inc., Seoul, South Korea).

### *De novo* assembly and DEG analysis of the transcriptome

We generated a transcriptome by *de novo* assembly of the mRNA sequences from the five varieties of asparagus and compared the genes involved in rutin and protodioscin biosynthetic pathways in green and white asparagus varieties cultivated under different light conditions. Briefly, the RNA-seq data generated by the Illumina platform were filtered by removing the adapter sequences and low-quality reads with a Phred quality score < 20 [21]. The clean sequencing reads were used for *de novo* assembly with the Trinity software v2.4.0. [22], which was operated using the default settings with a k-mer of 25 and a minimum contig length of 100. All contigs were defined as unigenes by the BLASTx analysis and were not extended. We focused on comparing the DEGs associated with the synthesis of the secondary metabolites, rutin and protodioscin between Atlas_W of white shoot asparagus (hereafter referred to as “Atlas_W”) and Atlas_G of green shoot asparagus (hereafter referred to as “Atlas_G”). DEGs were identified by estimating the relative transcript abundance, i.e., the reads aligned to all unique contigs. The aligned fragments were normalized using the fragment per kilo-base of exon model per million (FPKM) mapped reads method [23].

### Functional annotation and gene ontology (GO) classification

All unique contigs were used to identify candidate coding regions with TransDecoder (http://transdecoder.sourceforge.net). The candidate gene contigs were searched against the known genes by performing a BLASTx analysis in the National Center for Biotechnology Information (NCBI) non-redundant (nr) protein database. Other databases, including EGGNOG, KO (KEGG Orthology), and UniprotKB, were also used for gene annotation. Unigenes were identified based on high sequence similarity to the genes of other species at an E-value cutoff of 1.0E-05. The unigenes identified based on NR annotation were classified by a gene ontology (GO) analysis using the GOstats (http://www.bioconductor.org/packages/3.3/bioc/html/GOstats.html) and Blast2GO (http://www.genome.jp/kegg) programs. In the GO analysis, the genes were classified at the second level under the biological process, cellular components, and molecular function categories.

### cDNA synthesis and quantitative real-time PCR

cDNA was synthesized from 1 μg of total RNA using the PrimeScript RT Reagent Kit (Takara, Kusatsu, Japan). For all target genes and the *actin* gene (as an internal reference), the primers were designed with the PrimerQuest Tool of Integrated DNA Technologies (https://sg.idtdna.com) to conduct a quantitative real-time PCR (S1 Table). The quantitative real-time PCR was performed in a BIORAD CFX96 Real-time PCR system (Bio-Rad Laboratories, Hercules, CA, USA) with SYBR Premix Ex TaqII (Takara, Kusatsu, Japan) under the following conditions: pre-denaturation at 95°C for 15 min, 39 cycles of denaturation at 95°C for 20 s, annealing at 60°C for 40 s, and extension at 72°C for 20 s, with a final extension at 72°C for 10 min. The differences between the treatment means were evaluated using three independent replicates for each sample. Subsequently, the mean and standard deviation values of the three replicates of each sample were analyzed.

### High-performance liquid chromatography analysis

All chemicals used were of the analytical reagent grade and used as received. All solvents were purchased from Daejung Chemicals (Siheung, Korea), and the standards were purchased from Sigma-Aldrich (St. Louis, MO, USA). The Atlas green and white spear samples were stored at −70°C and freeze-dried for 48 h. Each sample was pulverized using a mortar and pestle. The powdered sample (0.1 g) was extracted with 1 mL of 70% methanol at 30°C for 24 h. The supernatant was collected by centrifugation at 14,000 rpm at 25°C for 10 min. After filtration using a 0.22-μm syringe filter (Woongki Science, Seoul, Korea), the filtrate was used for analysis. The sample was separated on a C18 column (250 mm × 4.6 mm, particle size 5 μm; Shimadzu, Kyoto, Japan) using a Prominence high-performance liquid chromatography (HPLC) system (Shimadzu, Kyoto, Japan) equipped with a diode array UV-vis detector. Solvent A was water (formic acid 0.1%) and solvent B was acetonitrile (formic acid 0.1%). The flow rate was 0.7 mL/min, the injection volume was 10 μL, and the column temperature was maintained at 40°C. The analytical wavelengths were 205 and 330 nm. Initially, the concentration of solvent B was 12%, which was then increased to 30% and 80% at 20 and 50 min, respectively.

### Statistical analysis

All analyses were repeated three times, and the data were expressed as means and standard deviations. The statistical analysis was performed using the SAS software (SAS 1998) and Duncan's multi-range test was performed with a significance level of *p* < 0.05.

## Results

### Illumina sequencing and *de novo* transcriptome assembly

We successfully obtained a complete transcriptome through *de novo* assembly of the Illumina RNA-seq reads from asparagus spears. A total of 340 311 532 paired-end reads were generated from the transcriptomes of five varieties of asparagus. The raw data can be accessed at GenBank NCBI, SRA PRJNA522348 (https://www.ncbi.nlm.nih.gov/sra/?term=PRJNA522348). After removing the adapter sequences, ambiguous reads, and low-quality reads, 336 599 498 high-quality clean reads were obtained with an average GC content of 47.824%. Among these reads, 41 218 018 were from Atlas (green), 42 688 134 were from Atlas (white), 53 569 526 were from Gijnlim, 50 989 974 were from Pacific purple, and 148 133 846 were from UC157 (Table 2). All clean reads were assembled into 239 873 contigs using the Trinity program, with an average contig length of 694 bp, a maximum contig length of 16 179 bp, and a minimum contig length of 201 bp. A short average contig length is normal in the *de novo* assembly using the Illumina high-throughput platform sequencing data in plants [24, 25]. The GC content of these contigs was 40.56% and 40.65% in all contigs and 181 710 unique transcriptomes, respectively (Table 3). A slightly higher than the GC ratio of the range of 39.1 ~ 39.5% depends of the chromosome sequence in the NCBI *Asparagus officinalis* annotation release 100 (https://www.ncbi.nlm.nih.gov/genome/10978). The largest contig of 16 179 bp was matched to the auxin transport protein BIG (5109AA, XP_010942266 in NCBI) in *Elaeis guineensis* through a BlastX search. The *de novo* assembly was performed by comparing the summary to the other case, mapping the cleaned reads back to the unigenes, using TransRate for quality assessment and manual search for a few contig sequences in the NCBI database. We defined 181 710 putative unique genes with a total size of 166 Mb in asparagus. These data are enough for gene annotation and for analyzing the genes associated with the biosynthesis of the secondary metabolites, rutin and protodioscin.

**Table 2:** Summary of transcriptome cDNA sequencing of the five varieties of asparagus.

**Raw data**					
Sample	Total (bp)	Reads	GC^1)^ (%)	> Q20 (%)	> Q30 (%)
Atlas_G	6 297 986 520	41 708 520	47.82	98.58	96.25
Atlas_W	6 500 499 566	43 049 666	47.94	98.78	96.72
Gijnlim	8 193 269 060	54 260 060	48.07	98.54	96.12
Pacific	7 786 078 534	51 563 434	48.34	98.59	96.21
UC157	22 609 207 652	149 729 852	47.98	98.81	96.97
**Trimmed data**					
Sample	Total (bp)	Reads	GC^1)^ (%)	> Q20 (%)	> Q30 (%)
Atlas_G	5 989 895 708	41 218 018	47.6	99.15	97.19
Atlas_W	6 142 507 132	42 688 134	47.77	99.26	97.52
Gijnlim	7 657 196 872	53 569 526	47.83	99.14	97.13
Pacific	7 446 001 383	50 989 974	48.2	99.15	97.15
UC157	21 099 688 742	148 133 846	47.72	99.34	97.86

^1)^ The ratio of guanine and cytosine bases in the DNA.

**Table 3:** Summary of *de novo* assembly of cDNA sequences from the transcriptomes of five varieties of asparagus.

	All contigs	Unique transcriptome
Total	239 873	181 710
GC content (%)	40.56	40.65
Maximum contig length (bp)	16 179	16 179
Average contig length (bp)	694.24	612.17
Total length (bp)	166 530 225	111 237 820
Genes annotated from NR Database	No execution	89 418

### Gene annotation

To identify putative genes, all assembled contigs were queried against various databases, including NR, EGGNOG, KO, and UniprotKB. The number of genes identified varied from 70 098 to 90 160, depending on the database (S2 Table). We identified a putative unigene set by performing a BLASTx analysis using public protein databases, including the NCBI NR protein database. In total, 89 418 genes were matched to the genes in the NR database. Among them, 79 896 unigenes had an E-value < 1e-5 (S3 Table). Furthermore, 102 140 contigs of the putative unigenes were found in the NR database through a BLASTx search. Asparagus could be identified as a plant with highly different genes lacking contig annotation as shown by a BLAST search using public databases. We next analyzed the species distribution of the matched genes. Among the numerous plant species, *E*. *guineensis* (16 813), followed by *Phoenix dactylifera* (13 770), *Musa acuminata* (5312), *Nelumbo nucifera* (1568), *Oryza sativa* (1171), and *Vitis vinifera* (1057), had the highest number of hits to the transcriptome of asparagus. Similar results have been reported by previous studies. In a study on sex expression mechanisms in asparagus, *E*. *guineensis* (6736), *P*. *dactylifera* (5515), *M*. *acuminata* (2695), and *O*. *sativa* (1853) had the highest number of sequence hits to asparagus unigenes [26].

**Fig 1:**
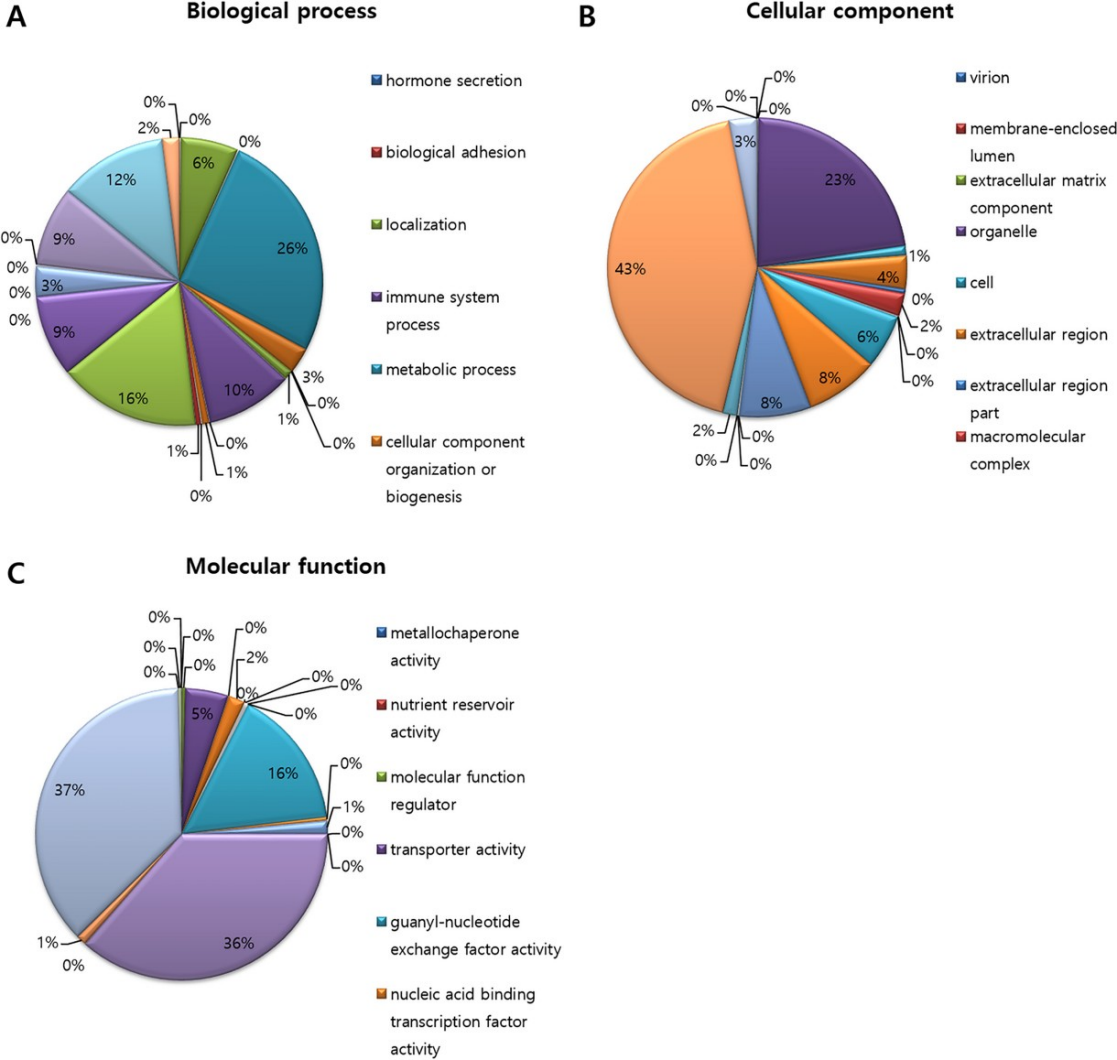
GO analysis of asparagus. (A: biological process, B: cellular component, C: molecular function).

Based on the BLAST results against the NR and KO databases, the sequences were annotated and classified into GO classes, including biological processes, cellular components, and molecular functions. In total, 36% of the genes were classified, with 30 106 (12.55%), 24 163 (10.07%), and 23 215 (9.68%) genes included under the biological process, cellular components, and molecular function categories, respectively (Fig 1). In the biological process category, metabolic process (7820), unclassified (4829), and biological regulation (3667) were the prominently represented sub-categories. Under the cellular components category, cell part (10 447) and organelle (5455) were the most highly represented sub-categories. Under the molecular function category, the highest proportions of genes were clustered under the catalytic activity (8597), binding (8459), and unclassified (3636) sub-categories.

### Differentially expressed genes among green and white asparagus

**Fig 2:**
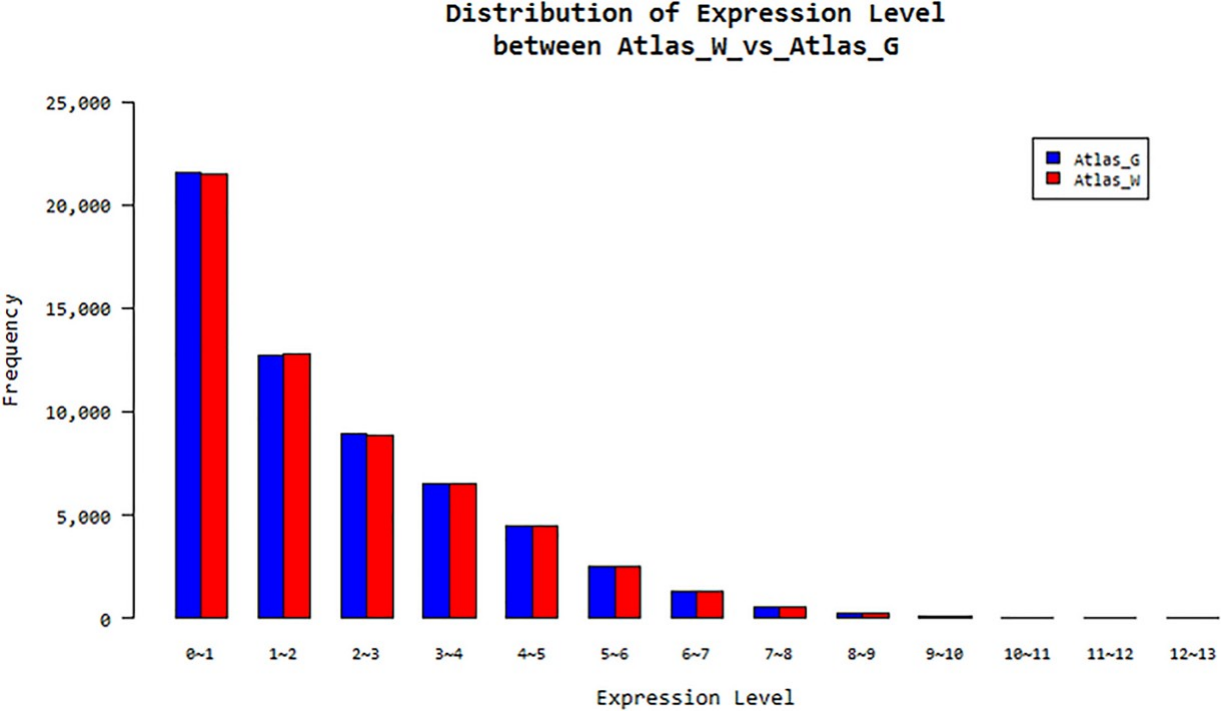
Distribution of expression levels between Atlas_G of green shoot asparagus and Atlas_W of white shoot asparagus.

The expression levels showed similar ratios in Atlas_G and Atlas_W in terms of the FPKM values ranging from 0 to 13 (Fig 2). A DEG analysis revealed that a total of 58 932 genes were differentially expressed between Atlas_G and Atlas_W. A specific gene responsible for functional material determination or metabolite differentiation should not exhibit a similar expression pattern. Therefore, we identified genes with different expression patterns between Atlas_G and Atlas_W for further analysis. Among 58 932 DEGs, 29 442 exhibited higher expression in Atlas_G than in Atlas_W, and 1951 DEGs showed a greater than four-fold difference in their expression levels. In Atlas_W, 29 490 DEGs exhibited higher expression, and 2 214 DEGs showed a greater than four-fold difference in expression compared with Atlas_G. The metabolic genes of rutin and protodioscin were searched using the NCBI NR database. Fifty-seven genes related to rutin (flavonoid) biosynthesis were identified, 12 of which showed more than four-fold differences in expression between Atlas_G of and Atlas_W. Fifty genes related to protodioscin (terpenoid) biosynthesis were found, of which two showed more than four-fold differences in expression between Atlas_G and Atlas_W (S4 Table).

### Gene expression associated with the synthesis of flavonoids and terpenoids in Atlas_G and Atlas_W

The biosynthesis of flavonoid compounds, such as rutin, starts with phenylalanine [27]. The rutin biosynthetic pathway leads to naringenin through p-coumaric acid, and then dihydrokaempferol, dihydromyricetin, and dihydroquercetin. Subsequently, quercetin and rutin are biosynthesized in the dihydroquercetin pathway.

**Fig 3:**
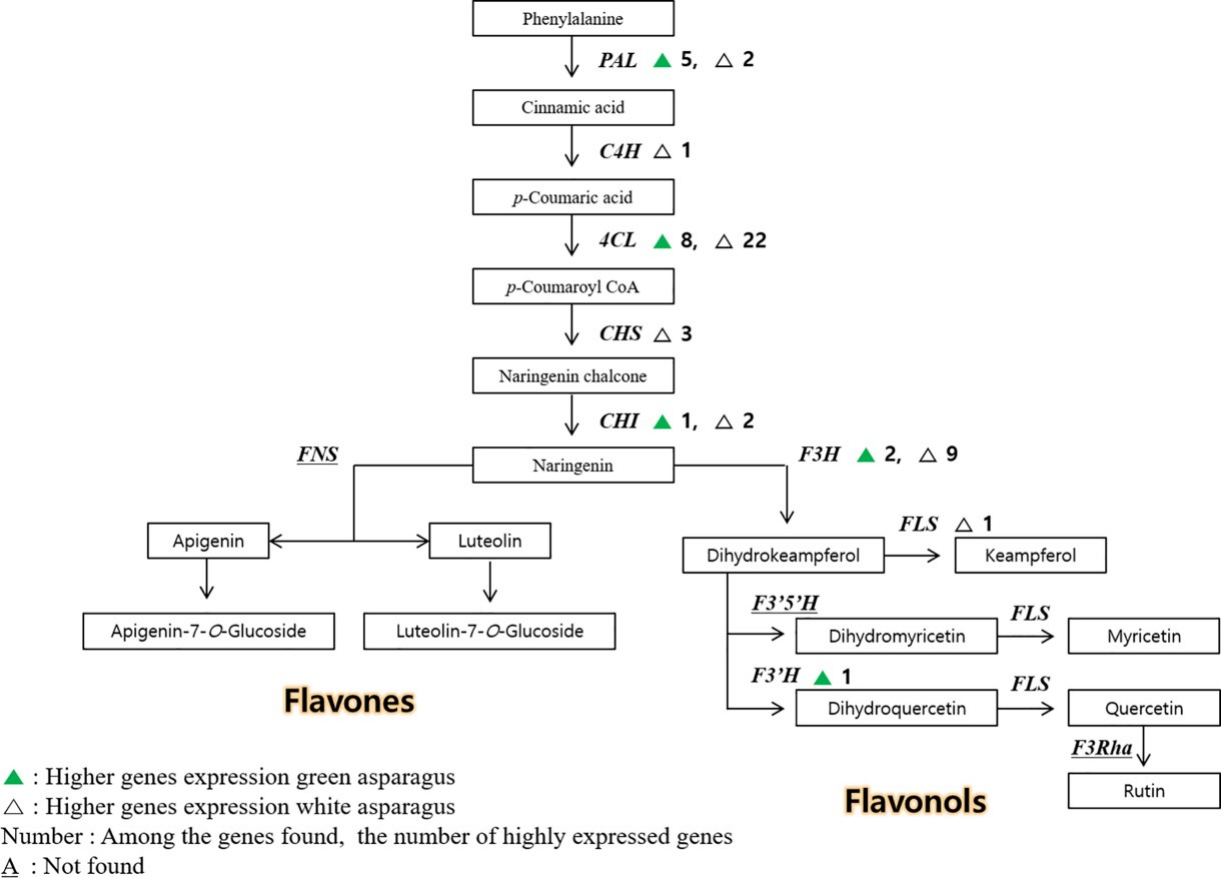
Biosynthetic pathways and compartmentalization of flavonoid synthesis in plants. 4CL, 4-coumarate:CoA ligase; C4H, cinnamate-4-hydroxylase; CHS, chalcone synthase; CHI, chalcone isomerase; F3H, flavanone 3-hydroxylase; F3′H, flavonoid 3′-hydroxylase; F3′5′H, flavonoid 3′,5′-hydroxylase; F3Rha, flavonol-3-O-glucoside l-rhamnosyltransferase; FLS, flavonol synthase; FNS, flavone synthase; PAL, phenylalanine ammonia-lyase. The numbering of the 3′, 4′, and 5′ carbon positions is shown in the structure. Enzyme names are in italics.

Through transcriptome analysis, we identified 17 and 40 upregulated genes related to flavonoid biosynthesis in Atlas_G and Atlas_W, respectively (Fig 3). Furthermore, among the enzyme genes involved in biosynthesis, the *F3′H* gene was found only in Atlas_G, and the *C4H* and *CHS* genes were found only in Atlas_W. The actual rutin content of Atlas_G was higher than that of Atlas_W (S1 Fig). Therefore, the genes with high expression levels in Atlas_G could play an important role in rutin biosynthesis. However, the genes encoding the enzymes involved in subsequent processes require further research.

Protodioscin is a representative triterpenoid of *A*. *officinalis* and belongs to the steroidal sapogenin family. Steroidal sapogenins are synthesized from cholesterol in several plants [28], and terpenoid compounds are divided into the mevalonate (MVA) and non-mevalonate (MEP) pathways. The MVA pathway begins with condensation of acetyl-CoA to acetoacetyl-CoA, which is then converted to isopentenyl diphosphate (IPP) through 3-hydroxy-3-methylglutaryl-CoA (HMG-CoA) and mevalonate (MVA). IPP acts as a precursor of triterpenoids. The other synthetic pathway, the MEP pathway, begins with the production of 1-deoxy-d-xylulose 5-phosphate (DXP) from pyruvate and glyceraldehyde-3-phosphate (G3P). In this process, IPP is produced via 2-C-methyl-d-erythritol 4-phosphate (MEP) and (E)-4-hydroxy-3-methylbut-2-enyl diphosphate (HMBPP) and then biosynthesized into triterpenoids.

**Fig 4:**
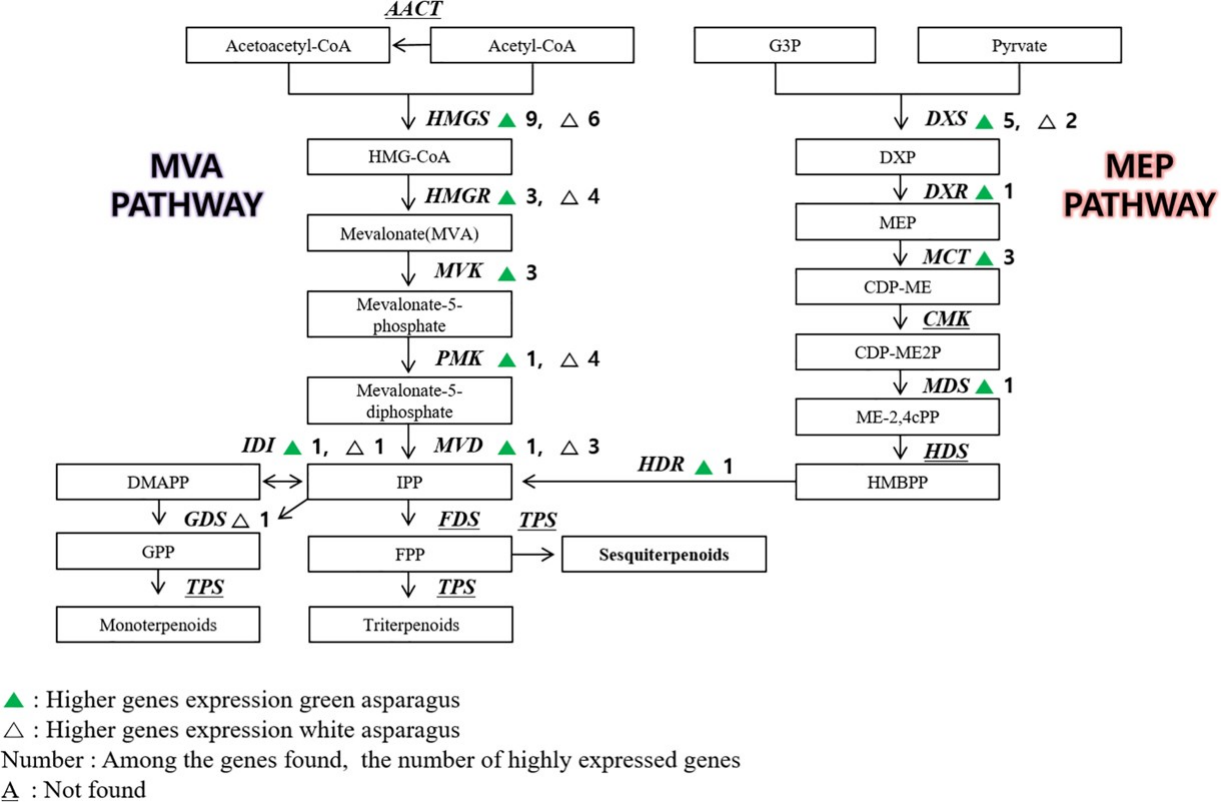
Biosynthetic pathways and compartmentalization of terpenoid synthesis in plants. AACT, acetoacetyl-CoA thiolase; AcAc-CoA, acetoacetyl-CoA; CDP-ME, 4-(cytidine 5′-diphospho)-2-C-methyl-d-erythritol; CDP-ME2P, 4-(cytidine 5′-diphospho)-2-C-methyl-d-erythritol phosphate; CMK, CDP-ME kinase; DMAPP, dimethylallyl diphosphate; DXP, 1-deoxy-d-xylulose 5-phosphate; DXR, DXP reductoisomerase; DXS, DXP synthase; FDS, farnesyl diphosphate synthase; FPP, farnesyl diphosphate; G3P, glyceraldehyde-3-phosphate; GDS, geranyl diphosphate synthase; GPP, geranyl diphosphate; HDR, (E)-4-hydroxy-3-methylbut-2-enyl diphosphate reductase; HDS, (E)-4-hydroxy-3-methylbut-2-enyl diphosphate synthase; HMBPP, (E)-4-hydroxy-3-methylbut-2-enyl diphosphate; HMG-CoA, 3-hydroxy-3-methylglutaryl-CoA; HMGR, HMG-CoA reductase; HMGS, HMG-CoA synthase; IDI, isopentenyl diphosphate isomerase; IPP, isopentenyl diphosphate; ISPS, isoprene synthase; MCT, 2-C-methyl-d-erythritol 4-phosphate cytidylyltransferase; MDS, 2-C-methyl-d-erythritol 2,4-cyclodiphosphate synthase; ME-2,4cPP, 2-C-methyl-d-erythritol 2,4-cyclodiphosphate; MEP, 2-C-methyl-d-erythritol 4-phosphate; MVD, mevalonate diphosphate decarboxylase; MVK, mevalonate kinase; PMK, phosphomevalonate kinase; TPS, terpene synthase. Enzyme names are in italics.

When the MVA and MEP pathways were examined, 29 genes were found to be highly expressed in Atlas_G, and 21 genes were highly expressed in Atlas_W (Fig 4). In addition, some genes, including *MVK*, *DXR*, *MCT*, *MDS*, and *HDR*, appeared only in Atlas_G. However, a comparison of the actual rutin and protodioscin content revealed that Atlas_W contained more protodioscin than Atlas_G (S2 Fig). Therefore, the genes highly expressed in Atlas_W might play an important role in the biosynthesis of terpenoids. However, the information on the enzymes that act after the MVA and MEP pathways is limited. We suggest that the enzymes involved in the subsequent processes might play an important role in terpenoid biosynthesis of asparagus, but this requires further study to confirm.

### Relationship of genes expressed during flavonoid and terpenoid biosynthesis

**Fig 5:**
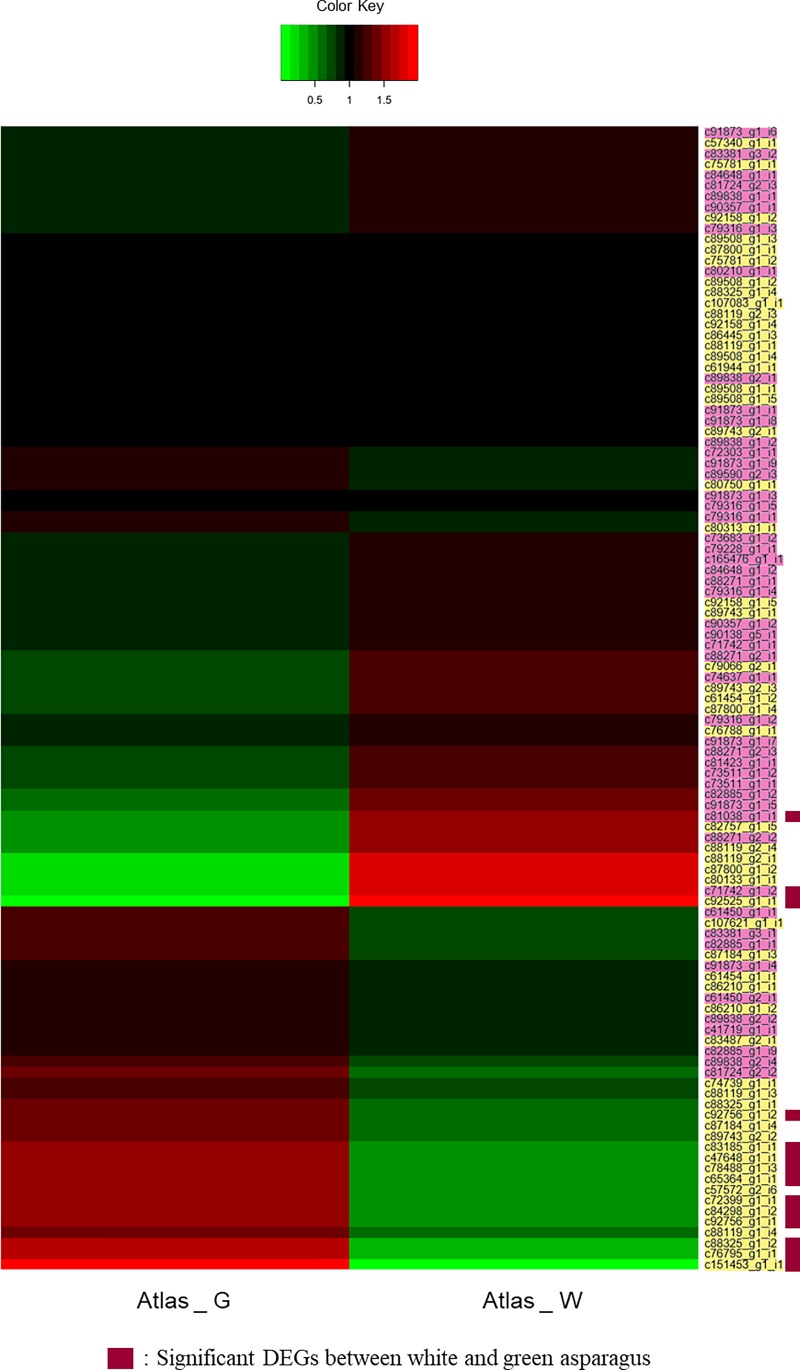
Heat map of expression differences in 57 flavonoid and 50 terpenoid biosynthesis genes of asparagus based on FPKM values (names in yellow represent flavonoid-related genes and names in pink represent terpenoid-related genes).

We identified significant DEGs involved in the biosynthesis of rutin and protodioscin (Table 4, Fig 5). The NR annotation suggested that four *4CL* genes and three *CHS* genes were similar to the genes of *Ornithogalum saundersiae*. Notably, these genes were all highly expressed in Atlas_W. This suggests that the biosynthetic pathways in Atlas_W and *O*. *saundersiae* are highly similar, and that this species might be helpful for studying the rutin and protodioscin biosynthesis of Atlas_W. However, the flavonoid rutin is not a major substance in Atlas_W (S1 Fig). Therefore, these genes might play a role in the biosynthesis of flavonoids other than rutin, and this information might contribute to the study of flavonoid biosynthesis in asparagus. Among the genes associated with asparagus flavonoid biosynthesis, the genes similar to the *4CL* gene of *P*. *dactylifera* had higher expression in Atlas_G. Furthermore, these genes showed the largest difference among the flavonoid genes of asparagus. As one of the main compounds of Atlas_G (S1 Fig), rutin is a flavonoid-based compound and, therefore, the expression of these genes might play an important role in the biosynthesis of rutin. Genes from *E*. *guineensis*, *P*. *dactylifera* and *Narcissus tazetta* were also associated with the flavonoid and terpenoid biosynthetic genes of asparagus that showed more than four-fold differences between Atlas_G and Atlas_W. In addition, the *E*. *guineensis* and *P*. *dactylifera* genes were found to match many asparagus genes in the gene annotation. Therefore, these genes were expected to have a great influence on the biosynthetic pathways in asparagus.

**Table 4:** The significant DEGs related to rutin and protodioscin biosynthesis between Atlas_W of white shoot asparagus and Atlas_G of green shoot asparagus.

Contig	Length	Enzymes	Atlas_W/Atlas_G log2 value	Atlas_G FPKM	Atlas_W FPKM
*c65364_g1_i1*	996	4-coumarate:CoA ligase-like protein [*Ornithogalum longibracteatum*]	2.804	16.52	48.67
*c72399_g1_i1*	791	4-coumarate:CoA ligase-like protein [*Ornithogalum longibracteatum*]	3.242	16.06	55.95
*c92756_g1_i1*	4201	4-coumarate:CoA ligase-like protein [*Ornithogalum longibracteatum*]	2.57	24.22	65.82
*c92756_g1_i2*	3261	4-coumarate:CoA ligase-like protein [*Ornithogalum longibracteatum*]	2.165	24.35	54.12
*c47648_g1_i1*	503	PREDICTED: 4-coumarate—CoA ligase-like 5 [*Elaeis guineensis*]	2.703	3.85	11.64
*c92525_g1_i1*	1752	PREDICTED: 4-coumarate—CoA ligase-like 1 [*Phoenix dactylifera*]	-12.321	19.41	0.4
*c76795_g1_i1*	1474	Chalcone synthase [*Ornithogalum saundersiae*]	4.526	26.9	137.58
*c83185_g1_i1*	2017	Chalcone synthase [*Ornithogalum saundersiae*]	2.835	30.87	95.92
*c151453_g1_i1*	1408	Chalcone synthase [*Ornithogalum saundersiae*]	11.338	0.07	10.85
*c78488_g1_i3*	1529	Chalcone isomerase 2 [*Narcissus tazetta var*. *chinensis*]	2.635	4.12	12.02
*c84298_g1_i2*	2357	Putative flavonol synthase/flavanone 3-hydroxylase [*Oryza minuta*]	2.66	8.92	24.98
*c88325_g1_i2*	1452	PREDICTED: flavonol synthase/flavanone 3-hydroxylase-like isoform X2 [*Phoenix dactylifera*]	2.202	0.41	1.86
*c71742_g1_i2*	626	Hydroxymethylglutaryl coenzyme A synthase [*Narcissus tazetta*]	-2.887	4.35	0.65
*c81038_g1_i1*	2633	PREDICTED: probable 1-deoxy-d-xylulose-5-phosphate synthase, chloroplastic [*Elaeis guineensis*]	-2.55	51.79	17.4

**Fig 6:**
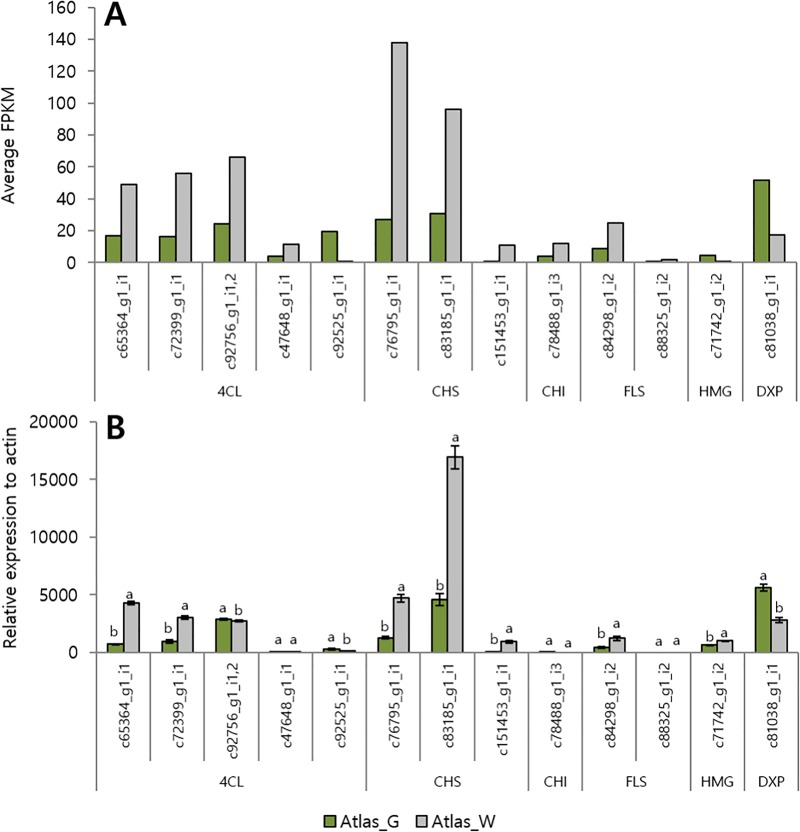
Comparison of the expression patterns of genes involved in flavonoid and terpenoid biosynthesis in Atlas_W of white shoot asparagus and Atlas_G of green shoot asparagus between the RNA-seq FPKM and qRT-PCR data. (A: RNA-seq FPKM data, B: qRT-PCR data). Values in a row with different letters are significantly different (*p* < 0.05), mean ± SD (*n* = 3).

The RNA-seq gene expression levels were screened using an absolute value of log2 fold change > 1 and FPKM > 1. A qRT-PCR was performed to validate the sequencing results. Primers were designed based on the alignments of the most conserved domains with the Integrated DNA Technologies database (http://www.sg.idtdna.com); *actin* was used as the reference gene. We tested the expression of 14 DEGs involved in flavonoid and terpenoid biosynthesis with qRT-PCR to validate the RNA-seq results (Fig 6). Nine of the 14 genes were similar to those of RNA-seq. Flavonoid-related genes showed high expression levels in Atlas_W according to the FPKM values. The expression levels of *c65364* and *c72399* were approximately 6.3 and 3.2 times higher in Atlas_W than in Atlas_G. The expression levels of *c76795*, *c83185* and *c84298* were 3.7, 3.6, and 2.8 times higher, respectively, in Atlas_W than in Atlas_G. In particular, *c151453* showed the largest difference; its expression level was 116.3 times higher in Atlas_W. However, some genes were hard to compare, including *c47648*, *c78488*, and *c88325*, because their expression levels were very low. The *c92756_g1_i1* and *c92756_g1_i2* nucleotide sequences were almost identical, making it difficult to distinguish them with the primers used. Therefore, the expression levels of both genes were combined. In contrast to the RNA-seq results, the *c92756* gene showed higher expression in Atlas_G than Atlas_W. For two terpenoid-related genes, *c81038* showed higher expression in Atlas_G according to the FPKM values, with approximately 2-fold difference, while *c71742* showed a higher expression level in Atlas_W in qRT-PCR in contrast to the FPKM values. In the case of *c92756* and *c71742*, the qRT-PCR results differed from the FPKM values, which was considered to be due to the correction of FPKM.

The growth condition that causes the greatest difference between Atlas_G and Atlas_W is light intensity. Therefore, the light intensity conditions can affect the expression levels of the biosynthetic genes of the secondary metabolites, rutin and protodioscin. These differences might contribute significantly to the differences of the rutin and protodioscin of Atlas_G and Atlas_W. The genes showing distinct differences in the rutin and protodioscin biosynthetic pathways were identified. The flavonoid- and terpenoid-related genes with expression differences greater than four-fold might be responsible for the differences in rutin and protodioscin between Atlas_G and Atlas_W. Thus, these genes might play an important role in the regulation of secondary metabolites, rutin and protodioscin in asparagus.

### Analysis of secondary metabolite content

**Fig 7:**
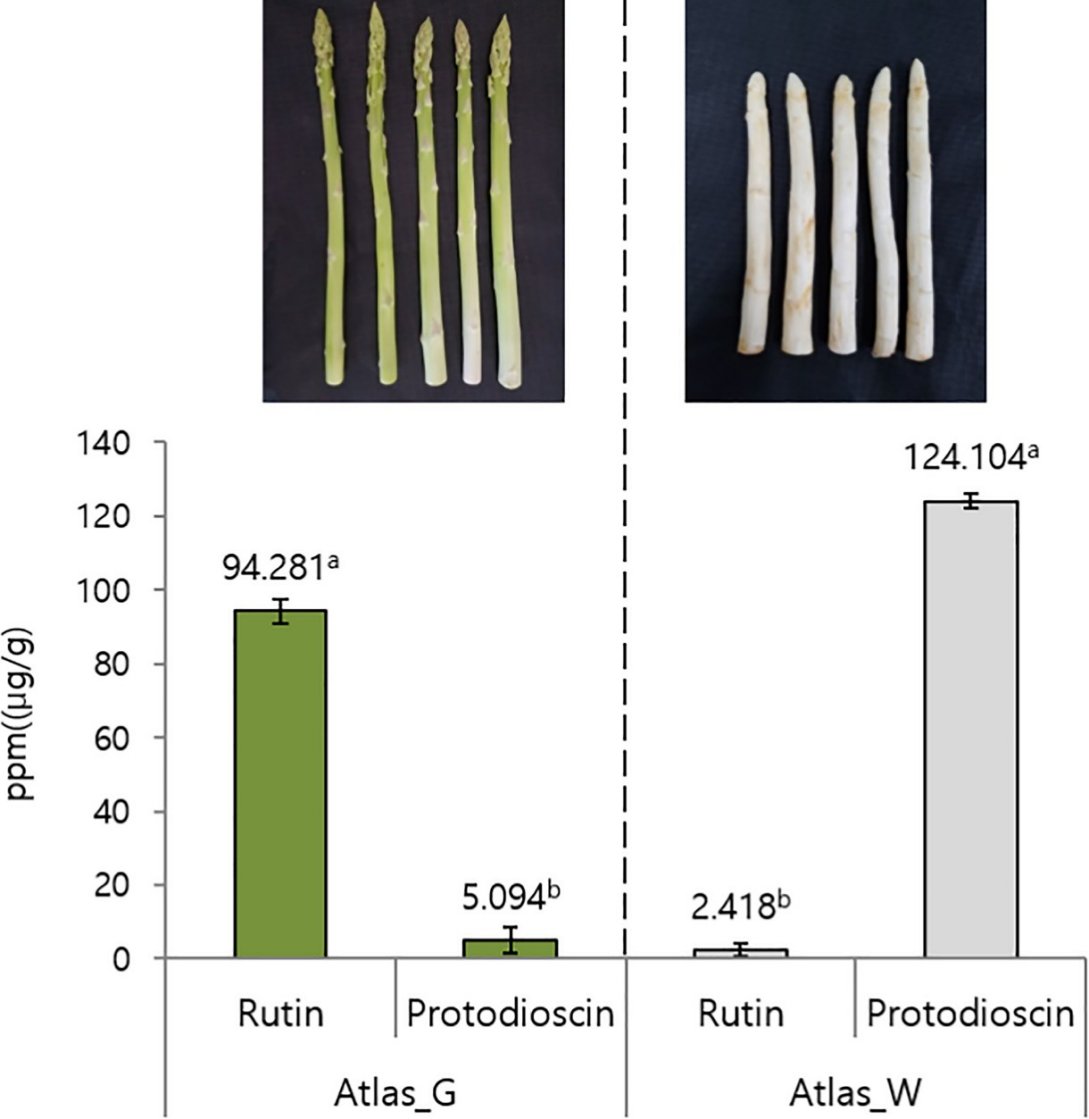
Rutin and protodioscin contents in Atlas_W of white shoot asparagus and Atlas_G of green shoot asparagus. Values in a row with different letters are significantly different (*p* < 0.05), mean ± SD (*n* = 3).

Gene expression and compound contents can vary significantly. Thus, a quantitative analysis of rutin- and protodioscin-based components of asparagus was conducted to investigate the secondary metabolite contents of asparagus. Rutin was detected at 330 nm and observed at 20.4 min (S1 Fig). Protodioscin was detected at 205 nm and observed at 30.3 min (S2 Fig). The quantitative analysis revealed that the rutin content in Atlas_G extract was 94.281 μg/g, while the protodioscin content was only 5.093 μg/g. By contrast, the protodioscin content in Atlas_W extract was 124.103 μg/g, which was significantly higher than in Atlas_G extract. However, the rutin content was 2.418 μg/g, which was significantly lower than in Atlas_G (Fig 7). These differences in metabolite contents between Atlas_G and Atlas_W are similar to those reported in previous studies [16, 17]. The analysis of secondary metabolites suggested that there were differences in gene expression between Atlas_G and Atlas_W. The expression analysis of the genes involved in the rutin biosynthetic pathway showed that Atlas_W had higher expression levels than Atlas_G of some genes. Conversely, among the genes involved in the protodioscin biosynthetic pathway, Atlas_G had higher expression levels than Atlas_W of some genes. When comparing these results with the contents of the secondary metabolites studied, the genes with higher expression levels in Atlas_G than in Atlas_W might be considered to be involved in rutin biosynthesis. In the case of protodioscin, the genes showing higher expression in Atlas_W than in Atlas_G might be considered to be involved in protodioscin biosynthesis.

## Discussion

### Genes involved in rutin and protodioscin biosynthesis

In our transcriptome analysis, we identified seven phenylalanine ammonia-lyase (*PAL)* genes, which are associated with phenylalanine and initiate flavonoid biosynthesis. The most frequently detected genes were the 4-coumarate:CoA ligase (*4CL)* genes; among the 57 flavonoid-related biosynthetic genes found, 30 were *4CL* genes. The *4CL* gene encodes an enzyme involved in the biosynthesis of p-coumaroyl CoA from p-coumaric acid, and has been reported to be associated with the expression of *PAL* genes [29]. Flavonol compounds, to which rutin belongs, are biosynthesized from naringenin and dihydrokaempferol. Eleven *F3H* genes related to the biosynthesis of naringenin and dihydrokaempferol were found. However, the information about the biosynthetic genes involved in subsequent steps is very limited. There are only two reports of the biosynthetic genes for quercetin or dihydroquercetin, which are directly related to rutin.

In *Fagopyrum esculentum*, which has a high rutin content, transcript sequencing was performed to observe the rutin accumulation and variation in seeds among varieties [30]. Similar to our study, the expression levels of the *PAL*, *C4H*, *4CL*, *CHS*, *CHI*, *F3H*, *F3*′*H*, and *FLS* genes were compared and analyzed. As a result of this analysis, it was reported that the expression level of the *FLS1* gene was related to rutin accumulation in *F*. *esculentum*. This suggests that the expression levels of the *PAL*, *C4H*, *4CL*, *CHS*, *CHI*, *F3H*, and *FLS* genes are important for rutin and flavonoid contents, but the authors highlighted the importance of the *FLS1* gene. However, asparagus has very limited information on related biosynthetic genes in the pathway after naringenin and the *F3H* genes. There are only two reports of the biosynthetic genes for quercetin or dihydroquercetin, which are directly related to rutin.

There are two terpenoid biosynthetic pathways, the MVA pathway and the MEP pathway. The MVA pathway begins with acetyl-CoA becoming HMG-CoA via HMGS. We found 34 genes in the MVA pathway to IPP, among which the *HMGS* genes were the most frequent. The other biosynthetic pathway, the MEP pathway, begins with DXS enzymes that biosynthesize DXP. We found 13 genes in the MEP pathway to IPP, among which the *DXS* genes were the most frequent. However, the information on biosynthesis steps after IPP is very limited. Only three terpenoid biosynthetic enzyme genes acting after IPP were found, and no information was found about the biosynthetic enzyme genes leading to the synthesis of triterpenoids, including protodioscin.

The terpenoid metabolism pathways in *Chlorophytum borivilianum* and *Valeriana fauriei* roots have been reported [31, 32]. The analysis of their terpenoid metabolic pathways focused on the genes related to the MVA and MEP pathways. At present, reports on protodioscin are very rare. Therefore, we had to analyze the metabolism of terpenoids, a group to which protodioscin belongs, and it was dependent on the information available for the genes involved in the MVA and MEP pathways.

For both flavonoid and terpenoid biosynthetic pathways, a little information is available on the posterior biosynthetic enzymes, necessitating further studies on the biosynthetic enzymes for plant secondary metabolites. In our asparagus transcriptome analysis, many potential genes were not annotated. Studies of these unannotated genes will be necessary to identify the biosynthetic enzyme genes in asparagus. Such studies might contribute to the study of secondary metabolite biosynthesis in other plants.

### Gene expression and actual secondary metabolite contents

Comparative genomics is a powerful tool for determining the genetic basis of biological functions. Forty of the 57 flavonoid biosynthetic genes identified in this experiment showed higher expression in Atlas_W than in Atlas_G. In addition, five *4CL*, three *CHS*, one *CHI*, and two *FH3* genes showed more than four times higher expression in Atlas_W than in Atlas_G. However, the rutin content of Atlas_G was 18 times higher than that of Atlas_W in this study. Gene expression can be activated by the sensing of environmental stresses, such as wounding and UV light irradiation [33, 34]. Because phenylpropanoid compounds can protect against these stresses, such stresses can promote their biosynthesis. Therefore, environmental stress in white asparagus cultivated under light-blocked conditions might increase the expression of the phenylpropanoid genes. In the case of rutin, the more light at the site, the more active the biosynthesis [17]. However, in white asparagus, the light is blocked and rutin biosynthesis is not performed efficiently. Further studies are needed to determine the roles of the highly expressed flavonoid-related genes in white asparagus.

Of the 50 genes involved in terpenoid biosynthesis, 29 genes showed higher expression in Atlas_G than in Atlas_W. Among these terpenoid biosynthetic enzyme genes, one *HMGS* gene and one *DXS* gene showed four times greater expression in Atlas_G. However, the protodioscin content was 60 times higher in Atlas_W than in Atlas_G in this study. A study of *Achyranthes bidentata* suggested that triterpenoid saponins are first synthesized in the leaves and then moved to the stem and root [35]. Experiments with leaves and root tissues of *Chlorophytum borivilianum* showed that the initial reaction of the terpenoid biosynthetic pathway occurs in the leaves [36]. This might explain the high expression levels of the terpenoid genes in Atlas_G used in this study. However, further studies are needed to determine the exact roles of these genes.

### Difference in gene regulation due to light conditions

The occurrence of green and white shoot asparagus is due to light conditions, in which the absence of light results in the formation of white shoot asparagus. There is a big difference in biological systems, including genetic and epigenetic factors related to the environmental stress of light.

Through the transcriptome analysis, we showed that the gene expression in green asparagus is more affected by light than that in white asparagus. Many light-related genes, such as light-regulated protein, photosystem I reaction center subunit, and chlorophyll A/B binding protein, were highly expressed in green asparagus. These genes are expressed in the chloroplasts of a cell, and their expression level vary according to the light conditions [37]. They play a role in photosystem-related complex formation and interaction stabilization [38] and affect plant development and growth through the synthesis of chlorophyll [39].

The difference in light conditions affected the expression of the genes related to the amino acid synthesis [40, 41]. Amino acids, such as glutamate and glutamine, have been reported to be affected by light and have important effects on hormone and secondary metabolite biosynthesis [42, 43]. The expression levels of the glutamate synthase and glutamine synthetase genes were compared among the genes analyzed in this experiment. A total of 12 glutamate synthase genes were detected, and they were highly expressed in Atlas_W. In addition, one of the glutamate synthase genes showed four times or more difference in expression. A total of seven glutamine synthetase genes were found, of which five genes were highly expressed in Atlas_G. Of these five genes, two showed more than 4-fold difference in expression.

Therefore, the difference in secondary metabolite abundance between white and green asparagus spears according to the light conditions is complex in biological systems, including genetic and epigenetic factors. The genetic factors, including DEGs involved in the biosynthesis of glutamate, can lead to the biosynthesis of the light-induced amino acids. The further effects of epigenetic factors should be studied to understand the difference of metabolite abundance between the white and green asparagus spears resulting from the difference in light conditions.

## Conclusion

*De novo* transcriptome sequencing was performed to identify functional differences between green and white asparaguses. A total of 239 873 sequences were assembled in an asparagus spear, and 89 418 distinct sequences were successfully annotated using the NR database. One hundred and seven genes were thought to be involved in the biosynthesis of rutin and protodioscin, which were the representative functional materials of green and white asparaguses. Among them, 14 genes showed a four-fold difference or more in their expression levels. Furthermore, the content of rutin and protodioscin was significantly different between Atlas_W and Atlas_G. This suggests that growing asparagus spears under different light conditions affects the expression of genes related to rutin and protodioscin biosynthesis, thus affecting the content of these compounds. Comparative analysis of the secondary metabolite content and gene expression levels can help in understanding the biosynthesis of functional compounds in green and white asparaguses. This research is expected to contribute to molecular, biological, and natural chemical studies of asparagus in the future.

## Supporting information

S1 Table Primer list for qRT-PCR. (XLSX)

S2 Table Gene annotations based on the NR, EGGNOG, KO and UniprotKB databases. (XLSX)

S3 Table The putative unigene set identified by BLASTx analysis using public protein databases, including the NCBI NR protein database. (XLSX)

S4 Table List of 57 flavonoid and 50 terpenoid biosynthesis genes identified based on FPKM values in asparagus. (XLSX)

S1 Fig HPLC chromatograms of the rutin standard and methanol extracts of freeze-dried white and green asparagus spears at 330 nm. (TIF)

S2 Fig HPLC chromatograms of the protodioscin standard and methanol extracts of freeze-dried white and green asparagus spears at 205 nm. (TIF)

## Abbreviations

DEG: differentially expressed gene
NR: NCBI non-redundant protein
qRT-PCR: Real Time reverse transcription polymerase chain reaction
4CL: 4-coumarate:CoA ligase
C4H: cinnamate-4-hydroxylase
CHS: chalcone synthase
CHI: chalcone isomerase
F3H: flavanone 3-hydroxylase
F3′H: flavonoid 3′-hydroxylase
F3′5′H: flavonoid 3′,5′-hydroxylase
F3Rha: flavonol-3-O-glucoside l-rhamnosyltransferase
FLS: flavonol synthase
FNS: flavone synthase
PAL: phenylalanine ammonia-lyase
AACT: acetoacetyl-CoA thiolase
AcAc-CoA: acetoacetyl-CoA
CDP-ME: 4-(cytidine 5′-diphospho)-2-C-methyl-d-erythritol
CDP-ME2P: 4-(cytidine 5′-diphospho)-2-C-methyl-d-erythritol phosphate
CMK: CDP-ME kinase
DMAPP: dimethylallyl diphosphate
DXP: 1-deoxy-d-xylulose 5-phosphate
DXR: DXP reductoisomerase
DXS: DXP synthase
FDS: farnesyl diphosphate synthase
FPP: farnesyl diphosphate
G3P: glyceraldehyde-3-phosphate
GDS: geranyl diphosphate synthase
GPP: geranyl diphosphate
HDR: (E)-4-hydroxy-3-methylbut-2-enyl diphosphate reductase
HDS: (E)-4-hydroxy-3-methylbut-2-enyl diphosphate synthase
HMBPP: (E)-4-hydroxy-3-methylbut-2-enyl diphosphate
HMG-CoA: 3-hydroxy-3-methylglutaryl-CoA
HMGR: HMG-CoA reductase
HMGS: HMG-CoA synthase
IDI: isopentenyl diphosphate isomerase
IPP: isopentenyl diphosphate
ISPS: isoprene synthase
MCT: 2-C-methyl-d-erythritol 4-phosphate cytidylyltransferase
MDS: 2-C-methyl-d-erythritol 2,4-cyclodiphosphate synthase
ME-2: 4cPP, 2-C-methyl-d-erythritol 2,4-cyclodiphosphate
MEP: 2-C-methyl-d-erythritol 4-phosphate
MVD: mevalonate diphosphate decarboxylase
MVK: mevalonate kinase
PMK: phosphomevalonate kinase
TPS: terpene synthase
