# Supplementary figures and images for: Transcriptome analysis of Asparagus officinalis reveals genes involved in the biosynthesis of rutin and protodioscin
Source: PLoS One. 2019 Jul 22;14(7):e0219973. doi: 10.1371/journal.pone.0219973 (PMC6645489; doi:10.1371/journal.pone.0219973)

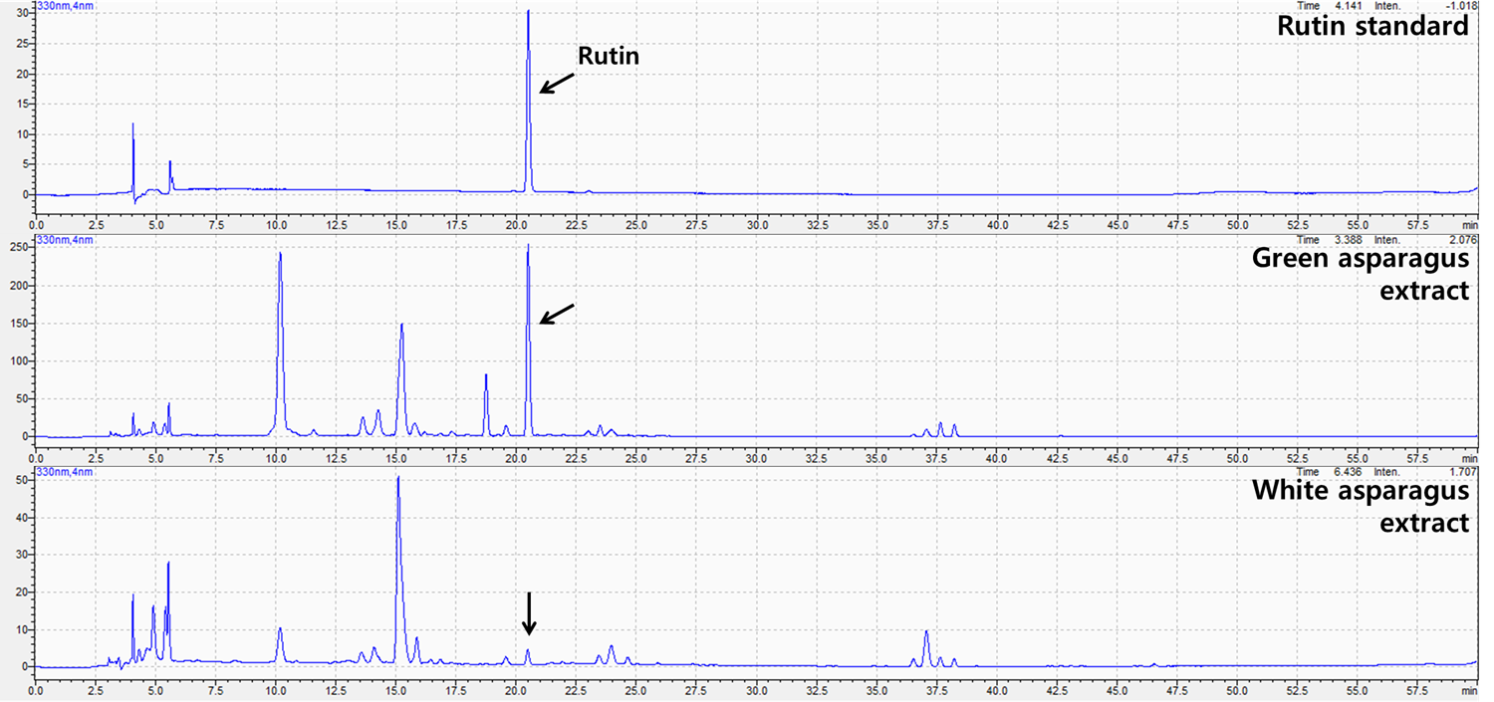

Supplement: S1 Fig — (TIF) [file pone.0219973.s005.tif]

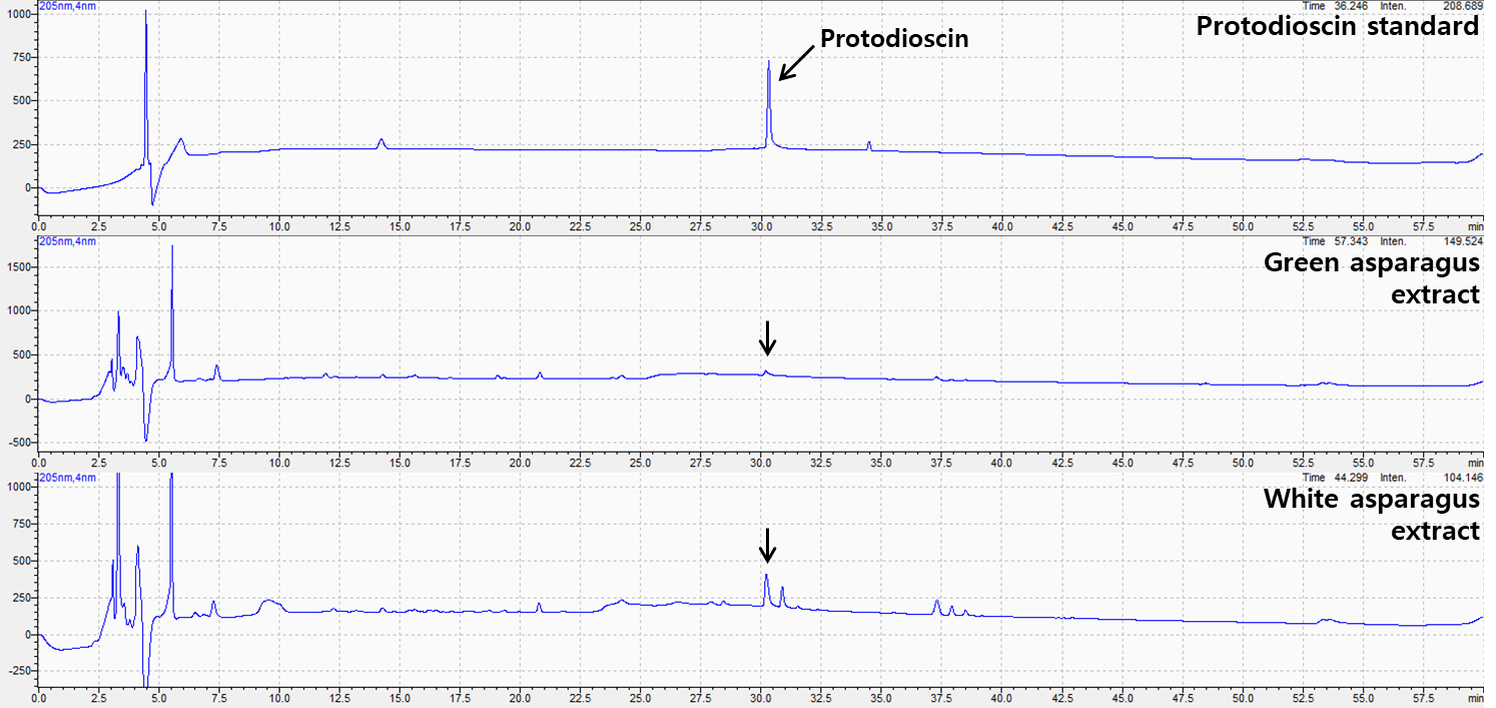

Supplement: S2 Fig — (TIF) [file pone.0219973.s006.tif]
